# Vascular involvement in Behçet’s disease: the immunopathological process

**DOI:** 10.1590/1677-5449.200170

**Published:** 2021-07-05

**Authors:** Raquelle Machado de Vargas, Maria Luiza Nunes da Cruz, Maria Paula Hashimoto Giarllarielli, Beatriz Mota Sano, Geovana Idelfoncio da Silva, Karina Furlani Zoccal, Cristiane Tefé-Silva

**Affiliations:** 1 Centro Universitário Barão de Mauá, Ribeirão Preto, SP, Brasil.

**Keywords:** Behçet syndrome, HLA-B51 antigen, systemic vasculitis

## Abstract

Behçet’s disease is a rare form of systemic vasculitis that affects small to large vessels. It is characterized by mucocutaneous, pulmonary, cardiovascular, gastrointestinal, and neurological manifestations. Its clinical presentation is quite wide, ranging from milder cases to severe cases, with multisystemic involvement, characteristically with exacerbations and remissions. Its etiopathogenesis is still unclear, although there is evidence of genetic, environmental, and immunological factors, such as the association with the HLA-B51 allele. In conjunction, all of these point to an abnormal immunopathological process, with activation of cells of innate and adaptive immunity, such as NK cells, neutrophils, and T cells, which generate specific response patterns and cytokines capable of generating mediators that can damage and inflame blood vessels, resulting in venous and arterial occlusions and/or aneurysm formation.

## INTRODUCTION

Behçet’s disease (BD) is a complex, multisystemic, chronic inflammatory syndrome of unknown etiology that was first described by the Turkish dermatologist Hulusi Behçet in 1937. From a clinical point of view, it is a vasculitis that presents with a variety of phenotypes, involving small to large vessels and characterized by recurrent mouth ulcers and other manifestations that include pulmonary, cardiovascular, gastrointestinal, and neurological conditions.^1^ Behçet’s disease has global distribution, but the majority of cases are found along the “silk road” trade route that extends from Asia to the Mediterranean.^1^ It has higher prevalence among males, particularly during the third and fourth decades of life.^2^

One third of patients have the vascular form of BD (vascular BD) and venous system involvement is more common than arterial involvement, primarily manifesting as deep venous thrombosis in the lower limbs. Vascular BD can involve small and large veins and arteries of the pulmonary circulation, which are common sites of aneurysm development, and it can also involve other pulmonary structures such as the parenchyma and pleura. The prevalence of pulmonary involvement in BD varies from 1 to 8%, and can manifest clinically with alveolar hemorrhage, pleural effusion, emboli, and pulmonary hypertension, while local rupture of aneurysms is one of the main causes of death of patients with BD.^3^^,^^4^

Behçet’s disease with central nervous system involvement (neuro Behçet) can manifest with venous sinus thrombosis, aneurysms, paralysis of cranial nerves, neuropsychiatric symptoms, and parenchymatous disease, which accounts for around 80% of cases, secondary to small vessel vasculitis involving the cerebral hemispheres and trunk and spinal cord.^1^^,^^2^ Gastrointestinal involvement in BD (gastro Behçet) is similar to gastrointestinal inflammatory diseases, with manifestations such as abdominal pains, diarrhea, and digestive hemorrhage.^2^ Cardiac injuries include pericarditis, thrombosis, acute myocardial infarction, and endomyocardial fibrosis. Characteristically, BD also involves the eyes, causing posterior uveitis, vascular thrombosis, and optic neuritis.^2^

Diagnosis of BD is clinical. In 1990, a set of diagnostic criteria were suggested by the International Study Group (ISG).^5^ In addition to an obligatory criterion of recurrent oral ulcers (at least three episodes in 12 months), the criteria include the following: recurrent genital aphthous ulcers; ocular lesions such as anterior or posterior uveitis; cutaneous manifestations such as erythema nodosum, pseudofolliculitis, papulopustular lesions or acneiform nodules; and a positive pathergy test.^5^ These criteria were revised, giving rise to the International Criteria for Behçet Disease (ICBD) in 2006, which included vascular manifestations in the BD criteria.^2^

Behçet’s Disease Current Activity Form (BDCAF) is a form comprising a range of questions for use when interviewing a patient who has already been diagnosed with BD in order to characterize disease activity. More recently, a simplified model of this protocol, the Behçet’s Disease Activity Index (BDAI), was proposed by the International Society for Behçet’s Disease. This form is designed to assess presence of a range of different manifestations of BD during the 4 weeks leading up to the interview, including clinical manifestations such as headaches, oral and genital ulcers, erythema nodosum, pustules, arthralgia, arthritis, abdominal pains/nausea/vomiting, digestive bleeding, ocular symptoms, central nervous system compromise, and large vessel compromise.^6^ The answers are used to produce an index ranging from zero to 12 that rating disease activity^6.^

The natural history of BD disease comprises periods of exacerbations and remissions and pathogenesis is still unclear, but there is evidence that tends to point to the same path: an abnormal immunopathological process with several triggers, such as infection, for example, associated with a genetic predisposition linked to the HLA-B51 allele.^2^ These triggers activate the immune system and initiate development of BD and subsequently initiate production of countless cytokines capable of generating the reactive cells responsible for vascular injuries.^7^

## METHODS

The study design is a bibliographic review of the literature. The review is based on domestic and international on-line articles published in English during the period from 2000 to 2020. The articles were accessed via the SciELO and PubMed databases using the following descriptors “Behçet’s disease”, “pathogenesis of Behçet’s disease” and “HLA-B51 gene”. The inclusion criteria were articles with full text available, published in Brazil or internationally, during the chosen period.

## RESULTS

**Figure 1:**
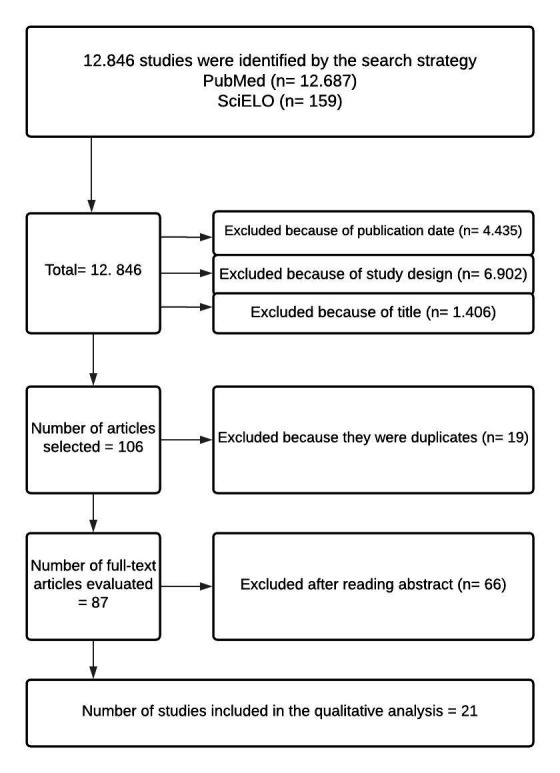
Flow diagram illustrating selection of articles.

Initially, a total of 12,687 references were identified on the databases. Application of the date limits, from 2000 to 2020, excluded 4,435 articles. Another exclusion criterion was study design, eliminating articles that were not reviews, systematic reviews, meta-analyses, or clinical trials, totaling 6,902 studies. A further 1,406 items were excluded after reading titles, selecting 106 articles. Nineteen of these were duplicates. The abstracts of the remaining 87 articles were read and only some of them met the inclusion criteria. The remainder (66) did not deal with the immunopathological process of BD or with vascular involvement. At the end of the selection process, just 21 publications were considered eligible, fitting the scope of the present article, as illustrated in Figure 1.

The meta-analyses included in the review were published by Zhang et al.^8^ and Maldini et al.^9^ The first of these encompassed a total of 16 articles involving 1,708 patients with BD and 1,910 healthy controls. Its objective was to assess the role played by polymorphisms of the gene coding tumor necrosis factor (TNF), a proinflammatory cytokine, in the pathogenesis of several inflammatory disorders, including BD. The second meta-analysis selected 78 articles that conducted a wide range of assessments of the relationships between clinical characteristics of BD and the HLA-B51 gene.

A systematic review by Deng et al.^10^ covered a number of genetic factors that may contribute to BD pathogenesis and the genes involved in immunological activation and regulation. Ahn et al.^11^ conducted a prospective, experimental, case-control study that assayed the profile of cytokines in aqueous humor and peripheral blood of BD patients with uveitis.

A number of retrospective studies (Alibaz-Oner et al.^12^ and Kechida et al.^13^) and comparative studies (Eksioglu-Demiralp et al.,^14^ Borhani Haghighi et al.,^15^ and Musabak et al.^16^) were also included, as shown in Table 1. The remainder were all review articles that met the inclusion criteria and were considered relevant to the subject of the present study since they covered introductory aspects of the disease, different clinical manifestations, and immunopathological features.

**Table 1:** Studies used in the review.

**Study**	**Objective**	**Details of the Study**	**Evidence grade (Oxford)**
Alibaz-Oner et al.^12^	To investigate therapeutic approaches during the initial event and relapses.	Patients with BD were enrolled (n = 936, mean age: 37.6±10.8) at 15 rheumatology centers in Turkey and classified according to the ISG criteria.	2C
Kechida et al.^13^	To describe the clinical characteristics of BD with cardiac and vascular involvement.	213 medical records were analyzed, from all patients with BD who met the ISGBD criteria seen from January 2004 to May 2016 at the Department of Internal Medicine.	3B
Eksioglu-Demiralp et al.^14^	To clarify the role played by neutrophils in the pathogenesis of BD.	Neutrophil activation was investigated in patients with BD using flow cytometry methods.	3B
Borhani Haghighi et al.^15^	To analyze cytokine profiles in the CSF of patients with neuro Behçet disease and viral meningitis.	IL-6, 8, 10, tumor necrosis-α, and interferon-γ were measured in CSF using the enzyme immunoassay method.	3B
Musabak et al.^16^	To investigate the relationship between serum IL-18 levels and BD disease activity and clinical presentations.	Sixty patients with BD and 20 healthy controls were included in the study. Patients were classified as having active or inactive disease.	3B

BD = Behçet’s disease; IL = interleukin; ISG = International Study Group; ISGBD = International Study Group for Behçet Disease; CSF = cerebrospinal fluid. 2C: ‘Outcomes’ research; 3B: Individual case-control study (Oxford Centre for Evidence-Based Medicine levels of evidence).

## DISCUSSION

Behçet’s disease is a systemic vasculitis characterized by painful and recurrent oral aphthous ulcers, genital ulcers, and ocular and cutaneous lesions (Table 2). Vascular involvement is observed in up to one third of cases and can affect vessels of all sizes, of both the arterial and venous systems, leading to occlusions and/or formation of aneurysms.^12^ The most frequently observed anatomopathological findings are infiltration of lymphocytes and neutrophils, which injure the vascular endothelium.

**Table 2:** Frequency of clinical lesions in Behçet’s disease.

	**Frequency (%)**
Oral lesions	95
Neurological manifestation	10-50
Gastrointestinal manifestation	5-60
Vascular manifestation	5-30
Cardiac manifestation	1-5
Pulmonary manifestation	1-8

**Figure 2:**
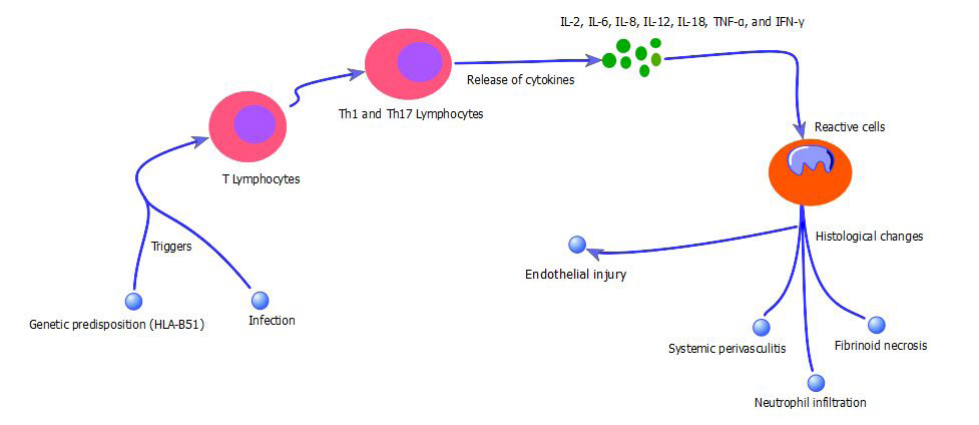
Diagram illustrating the immunopathological mechanism of Behçet’s disease. The HLA-B51 allele and infections mediate pathogenesis of Behçet’s disease. These triggers inappropriately activate an immune system capable of producing cytokines and, consequently, reactive cells, injuring vessels as a consequence. IL = interleukin; IFN = interferon; TNF = tumor necrosis factor.

The articles selected for this review were notably heterogeneous in relation to the causes of BD. Etiology is still unclear, although undoubtedly multifactorial, but can be classified into the three main and most important causes: genetic, inflammatory, and infectious. These three interrelated factors are shown in Figure 2, which illustrates the immunopathological mechanisms of BD.

### Genetic factor

Since the first reports emerged of a possible relationship between the HLA-B51 allele and BD, a series of studies have been conducted on the association, although the association remains indefinite.^2^ Many of these studies reported a higher prevalence of the HLA-B51 allele in patients with BD, in addition to association with more complex and severe expressions of the clinical manifestations and worse outcomes of ocular or neurological involvement.^10^ It was also demonstrated that the HLA-B51 allele predominates in men and is associated with moderately higher prevalence rates of genital ulcers and ocular and cutaneous manifestations, and with abnormalities of coagulation and in the endothelium, increasing the risk of development of aneurysms and thrombotic events.^9^^,^^12^

A retrospective study of data for 2004 to 2016 from the Department of Internal Medicine and Endocrinology at the Fattouma Bourguiba University Hospital, in Tunisia, showed that the allele was present in 15.92% of BD patients.^13^ The same study, which analyzed 213 patients, also reported important data on BD, such as presence of oral ulcers in 98.6% of the patients and 30% vascular involvement, showing their importance in diagnosis of disease.

However, genetic factors cannot entirely explain the pathogenesis of BD. The hypothesis that the HLA-B51 allele is linked to a range of environmental triggers, such as bacterial and viral infections, for example, which provoke exacerbation of the immune process and inflammatory response, appears to make more sense.^17^

However, the fact that the HLA-B51 allele is absent in around 80% of BD patients suggests that other factors must also be analyzed in relation to the genesis of BD.^10^^,^^18^

### Inflammatory and immunological process

The inflammatory response and the immunological process are unclear and complex and can trigger abnormalities affecting the coagulation cascade, causing endothelial injury, facilitating formation of thrombi, emboli, and aneurysms.^19^ Recently, immunopathogenesis and involvement of immune and adaptive cells and cytokines in genesis of BD have been discussed.^10^

Natural killer (NK) cells are the most important elements in innate immunity, since they do not only play a cytotoxic role in infected cells, but also regulate the functions of other immune system cells that secrete cytokines, including TNF‐α, interferon (IFN) γ and α, and interleukins (IL) 2, 4, and 10.^7^

Neutrophils also play a fundamental role in the innate immunoresponse and are the first line of defense against infectious diseases. Studies have demonstrated that one of the principal mechanisms in pathogenesis of BD is activation of neutrophils with increased chemotaxis and generation of superoxide radicals. Additionally, one study found evidence that BD patients’ neutrophils exhibited a high level of intrinsic activation, which may be associated with presence of the HLA-B51 gene, and are involved in perivascular infiltration of lesions in the vascular form of BD.^14^

Adaptive responses in BD involve T cells with Th1 and Th17 response patterns, which play an important role in pathogenesis of the disease.^7^ The Th1 cell-mediated immunoresponse plays a fundamental role in pathogenesis of BD and its prevalence is significantly higher in patients with active BD than in patients with inactive BD.^7^ Table 3 summarizes how these cells act in the pathogenesis of BD and also the cytokines involved.

**Table 3:** Principal cells involved in the pathogenesis of Behçet’s disease.

**NK cells**	The frequency of NK cells is reduced in autoimmune diseases and their cytotoxicity is impaired. Some studies have demonstrated low numbers of NK cells in patients with BD, compromising immunity.
**Neutrophils**	Neutrophils can damage host cells and tissues, making them a trigger factor of inflammation, which, in turn, triggers the immunoresponse.
**Th1 cells**	The Th1 cells that produce IFN-γ activate macrophages responsible for cell-mediated immunity to intracellular pathogens and associated with many organ-specific autoimmune diseases, including BD.
**Th17 Cells**	There is a significantly higher frequency of Th17 cells in circulation in patients with active BD than in the same patients when in remission.

BD = Behçet’s disease; IFN = interferon; NK = natural killer.

### Infections

Bacterial or viral infections can act as triggers for development of the disease in people who carry the HLA-B51 allele.^12^ The microorganisms that have been most associated with BD are the herpes simplex virus and *Streptococcus* ssp, but others have also been described, such as parvovirus B19, *Helicobacter pylori*, cytomegalovirus, Epstein-Barr virus, herpes zoster virus and *Staphylococcus aureus*.^2^^,^^7^

These infections are associated with systemic inflammation in genetically susceptible individuals, which may trigger an inappropriate and exacerbated immunoresponse and provoke endothelial injury. This process is illustrated in Figure 2.

### The role of inflammatory cytokines

The immunopathogenesis of BD has been widely studied and several immune system cells and cytokines may be involved, primarily those that are proinflammatory, accelerating impairment of endothelial function.^7^ One study reported that many of these inflammatory cytokines were found at elevated levels in serum from BD patients and, because of this evidence, TNF-α and IFN-α antagonists demonstrated good efficacy and have been adopted as agents for treatment of BD.^7^

As illustrated in Figure 2, there is initially an association between BD and the HLA-B51 allele, which demonstrates an increased risk of BD. Infectious agents can also act as triggers of an abnormal immunoresponse. Both can trigger hyperreactivity of T cells and neutrophils.^14^ T lymphocytes respond to viral and bacterial infections, tending to a Th1 and Th17 response pattern, which in turn produces proinflammatory mediators such as IL-2, IL-6, IL-8, IL-12, IL- 18, IL-21, TNF-α and IFN-γ.^7^ It is worth outlining how some of these are related to BD:

IL-6: excessive IL-6 production is related to autoimmune and chronic inflammatory diseases. Increased IL-6 has been reported in the cerebrospinal fluid of patients with neurological BD involvement.^15^

IL-18: plays an important role in immunoresponse of Th1 cells. The functions of IL-18 are to promote production of IFN-γ, activating NK cells, and to induce cytotoxic activity. IL-18 levels were higher in all patient subgroups when compared to healthy controls, demonstrating the correlation between this cytokine and disease activity.^16^

IFN-γ: the Th1 cells that produce IFN-γ activate macrophages and are associated with autoimmune diseases, including BD.^7^ In one study that assessed the role of cytokines in BD uveitis, it was found that IFN-γ levels in the aqueous humor of patients with BD were significantly higher than in that of patients with BD, but without uveitis.^11^

TNF-α: plays a fundamental role in induction and maintenance of inflammation in the autoimmune response, in addition to being involved in several physiological and pathological processes, such as initiation of inflammation, immunoregulation, and cell proliferation.^8^ In inflammatory diseases, TNF-α is primarily produced by macrophages, T cells, B cells, neutrophils, NK cells, and endothelial cells. Serum TNF-α levels are elevated in patients with active BD.^7^

IL-21: is a cytokine from the IL-2 family that promotes expansion of CD8^+^ effector T cells and can activate NK cells.^7^ There is evidence of the critical role of IL-21 in BD inflammatory injuries, promoting Th17 effector cells and suppressing regulator T cells, constituting a promising target for a new BD treatment.^20^

### Vascular involvement in BD

Vasculitis in BD is neutrophil-predominant, affecting all layers of the vessel and *vasa vasorum*, which can present fibrous thickening and nonspecific inflammatory infiltrate in the late phase.^5^ Associated with BD vasculitis, there is also hypercoagulability, with excessive thrombin formation, reduced fibrinolysis, platelet hyperactivity, and formation of platelet/neutrophil complexes.^2^ All of these changes can favor formation of thrombosis. The inflammatory process in the vessel wall can cause destruction of the elastic fibers, leading to transmural necrosis of the walls of major muscular arteries, with consequent formation of aneurysms.^5^

### Treatment

Although the traditional treatment options involve glucocorticoids and immunosuppressants, there is little evidence regarding their efficacy in BD.^1^ IFN and TNF-α antagonists have demonstrated good efficacy and are the first-line agents used to improve prognosis in BD. Treatment of patients using TNF-α antagonists (Infliximab, Etanercept, and Adalimumab) is based on control of the inflammatory response, which is effective in severe and refractory manifestations of BD.^7^ Treatment with IFN, primarily INF-α, has demonstrated benefits for habitual treatment of the disease, with effective antiviral, antitumoral, and immunomodulatory activities in management of BD.^1^ However, there is still a lack of data on the ideal therapeutic approaches and informative laboratory markers for monitoring disease progression are also lacking.^21^

## CONCLUSIONS

The pathogenesis of BD has not yet been fully elucidated. However, studies have demonstrated that certain triggers, such as bacterial and viral infections, and genetic predisposition (presence of the HLA-B51 allele) can initiate and interfere in development of BD, via exacerbated activation of the immune system, resulting in synthesis of countless cytokines capable of producing the reactive cells responsible for injury to vessels and hypercoagulability. However, more detailed studies are needed to better explain the mechanisms involved in the immunopathological process of BD.
