# Supplementary material for: Vascular involvement in Behçet’s disease: the immunopathological process
Source: J Vasc Bras. 2021 Jul 5;20:e20200170. doi: 10.1590/1677-5449.200170 (PMC8256877; doi:10.1590/1677-5449.200170)
Supplement: Translation

# Translation: Acometimento vascular na doença de Behçet: o processo imunopatológico

**Authors:** Raquelle Machado de Vargas, Maria Luiza Nunes da Cruz, Maria Paula Hashimoto Giarllarielli, Beatriz Mota Sano, Geovana Idelfoncio da Silva, Karina Furlani Zoccal, Cristiane Tefé-Silva

**Affiliations:** 1 Centro Universitário Barão de Mauá, Ribeirão Preto, SP, Brasil.

**Keywords:** síndrome de Behçet, antígeno HLA-B51, vasculite sistêmica

## Abstract

A doença de Behçet constitui uma forma rara de vasculite sistêmica, que acomete de pequenos a grandes vasos. É caracterizada por manifestações mucocutâneas, pulmonares, cardiovasculares, gastrointestinais e neurológicas. Sua apresentação clínica é bastante ampla, variando de casos mais brandos a casos graves, com acometimento multissistêmico, caracteristicamente com exacerbações e remissões. Suas causas ainda são desconhecidas; entretanto, há evidências genéticas, ambientais e imunológicas, como a associação com o alelo HLA-B51. Todas essas, em conjunto, apontam para um processo imunopatológico anormal, com ativação de células da imunidade inata e adaptativa, como as células *natural killer*, neutrófilos e células T, que geram padrões de respostas e citocinas específicos capazes de gerar mediadores que podem lesionar e inflamar o sistema vascular, resultando em oclusões venosas, arteriais e/ou formação de aneurismas.

## INTRODUÇÃO

A doença de Behçet (DB) é uma síndrome inflamatória complexa, multissistêmica, crônica e de etiologia desconhecida, descrita inicialmente pelo dermatologista turco Hulusi Behçet em 1937. Do ponto de vista clínico, é uma vasculite que apresenta vários fenótipos, acometendo pequenos a grandes vasos, caracterizada por úlceras orais recorrentes e outras manifestações, que incluem afecções pulmonares, cardiovasculares, gastrointestinais e neurológicas^1^. A DB tem distribuição mundial, mas a maioria dos casos é encontrada na chamada “rota da seda”, a qual se estende da Ásia ao Mediterrâneo^1^. A DB é mais prevalente no sexo masculino, principalmente entre a terceira e quarta décadas de vida^2^.

A forma vascular da DB (DB vascular) acomete até um terço dos pacientes, sendo o envolvimento do sistema venoso mais comum que o do arterial, manifestando-se principalmente por trombose venosa profunda de membros inferiores. A DB vascular pode envolver também pequenas e grandes veias e artérias da circulação pulmonar, que são focos comuns de desenvolvimento de aneurismas, além de outras estruturas pulmonares, como parênquima e pleura. A prevalência do envolvimento pulmonar na DB varia de 1 a 8%, podendo se manifestar clinicamente com hemorragia alveolar, derrame pleural, embolia e hipertensão pulmonar, sendo a rotura local de aneurismas uma das principais causas de morte nos pacientes portadores da DB^3^^,^^4^.

A DB com acometimento do sistema nervoso central (neuro Behçet) pode se manifestar como tromboses de seios venosos, aneurismas, paralisia de nervos cranianos, sintomas neuropsiquiátricos e doença parenquimatosa, o que representa cerca de 80% dos casos, secundária a vasculites de pequenos vasos que atinge o tronco, hemisférios cerebrais e medula espinal^1^^,^^2^. O acometimento gastrointestinal na DB (gastro Behçet) é semelhante ao das doenças inflamatórias gastrointestinais, com manifestações como dor abdominal, diarreia e hemorragia digestiva^2^. As lesões cardíacas incluem pericardite, trombose, infarto agudo do miocárdio e fibrose endomiocárdica. Caracteristicamente, a DB acomete também o olho, causando uveíte posterior, trombose vascular e neurite óptica^2^.

O diagnóstico da DB é clínico. Em 1990, um conjunto de critérios de diagnóstico foi sugerido pelo *International Study Group* (ISG)^5^. Esses critérios incluem, além do critério obrigatório de úlceras orais recorrentes (pelo menos três episódios em 12 meses), os seguintes: lesões aftosas genitais recorrentes; lesões oculares como uveíte anterior ou posterior; manifestações cutâneas como eritema nodoso, pseudofoliculite, lesões papulopustulares ou nódulos acneiformes; e o teste de patergia positivo^5^. Tais critérios foram revisados e, em 2006, deram origem ao *International Criteria Behçet Disease* (ICBD), que incluiu as manifestações vasculares entre os critérios da DB^2^.

O chamado *Behçet’s Disease Current Activity Form* (BDCAF) é um formulário com diversas questões apresentadas para entrevista do paciente com DB já diagnosticada, para caracterização da atividade da doença. Posteriormente, um modelo simplificado desse protocolo chamado de *Behçet's Disease Activity Index* (BDAI) foi apresentado pela Sociedade Internacional da DB. Esse formulário tem a finalidade de avaliar a presença de diferentes manifestações da DB nas 4 semanas anteriores à entrevista, que incluem diferentes manifestações clínicas, como: cefaleia, úlceras orais e genitais, eritema nodoso, pústulas, artralgia, artrite, dor abdominal/náuseas/vômitos, sangramento digestivo, sintomas oculares, comprometimento do sistema nervoso central e comprometimento de grandes vasos^6^. Assim, produz-se um índice, que varia de zero a 12, para avaliar a atividade da doença^6^.

A história natural da doença é composta por períodos de exacerbações e remissões, e sua patogênese é ainda incerta, mas há evidências que tendem para um mesmo caminho: um processo imunopatológico anormal é desencadeado, envolvendo diversos gatilhos, como infecção, por exemplo, associados à predisposição genética do alelo HLA-B51^2^. Esses gatilhos ativam o sistema imunológico e iniciam o desenvolvimento da DB e, na sequência, iniciam a produção de inúmeras citocinas capazes de gerar células reativas responsáveis pelas lesões vasculares^7^.

## MÉTODOS

Trata-se de uma pesquisa definida como revisão bibliográfica de literatura. O estudo foi realizado por meio de artigos on-line, nacionais e internacionais e na língua inglesa, cujo corte temporal foi o período entre 2000 e 2020. Os artigos foram retirados das seguintes bases de dados: SciELO e PubMed. Utilizaram-se os seguintes descritores: “doença de Behçet”, “patogenia da doença de Behçet” e “gene HLA-B51”. Os critérios de inclusão foram textos completos e disponíveis, publicados nacionalmente e internacionalmente no período determinado.

## RESULTADOS

Inicialmente, foram encontradas 12.687 referências nas bases de dados. Após o corte temporal de publicação, do ano 2000 até 2020, foram excluídos 4.435 artigos. Também foi um critério de exclusão artigos que não fossem de revisão, revisão sistemática, metanálise ou ensaio clínico, somando 6.902 estudos, e aqueles excluídos pelo título, totalizando 1.406. Foram selecionados, então, 106 artigos e eliminados aqueles em duplicidade. Os resumos dos 106 artigos foram lidos, sendo 19 deles excluídos por duplicidade e somente alguns enquadrados nos critérios de inclusão. Os demais (66) não abordavam o processo imunopatológico da DB ou acometimento vascular. Ao final, apenas 21 trabalhos foram considerados elegíveis por englobar o escopo de estudo do presente artigo, como resume a Figura 1.

**Figura 1:** Fluxograma de seleção de artigos.

As metanálises utilizadas foram a de Zhang et al.^8^ e a de Maldini et al.^9^. A primeira incluiu um total de 16 artigos, envolvendo 1.708 pacientes com DB e 1.910 controles saudáveis, com o objetivo de avaliar a associação entre polimorfismos do gene do fator de necrose tumoral (TNF), uma citocina pró-inflamatória na patogênese de vários distúrbios inflamatórios, incluindo a DB. Já a segunda selecionou 78 artigos que avaliaram de forma abrangente as relações entre as características clínicas da DB e o gene HLA-B51.

A revisão sistemática de Deng et al.^10^ abordou os vários fatores genéticos que podem contribuir para a patogênese da DB e os genes envolvidos na ativação e regulação imunológica. O estudo de Ahn et al.^11^ foi prospectivo, experimental e de caso-controle, que determinou o perfil de citocinas no humor aquoso e no sangue periférico de pacientes com uveíte portadores da DB.

Outros artigos incluíram estudos retrospectivos (Alibaz-Oner et al.^12^ e Kechida et al.^13^) e estudos comparativos (Eksioglu-Demiralp et al.^14^, Borhani Haghighi et al.^15^ e Musabak et al.^16^), conforme detalhado na Tabela 1. Os demais artigos utilizados foram artigos de revisão que se enquadraram nos critérios de inclusão e foram considerados relevantes no tema do presente estudo ao abordarem aspectos introdutórios da doença, diferentes manifestações clínicas e aspectos imunopatológicos.

**Tabela 1:** Estudos utilizados na revisão.

**Trabalho**	**Objetivo**	**Detalhes do Estudo**	**Grau de evidência (Oxford)**
Alibaz-Oner et al.^12^	Investigar as abordagens terapêuticas durante o evento inicial e recidivas.	Foram incluídos pacientes com DB (n = 936, idade média: 37,6±10,8), classificados de acordo com os critérios do ISG de 15 centros de reumatologia da Turquia	2C
Kechida et al.^13^	Descrever as características clínicas da DB com o envolvimento cardíaco e vascular.	Foram analisados 213 prontuários de todos os pacientes com DB acompanhados entre janeiro de 2004 e maio de 2016 no Departamento de Medicina Interna e que preencheram os critérios do ISGBD.	3B
Eksioglu-Demiralp et al^14^.	Esclarecer o papel dos neutrófilos na patogênese da DB.	A ativação de neutrófilos foi investigada em pacientes com DB por métodos de citometria de fluxo.	3B
Borhani Haghighi et al.^15^	Analisar perfis de citocinas no LCR de pacientes com doença neuro Behçet e meningite viral.	IL-6, 8, 10, fator de necrose tumoral-α e interferon-γ foram medidos no LCR usando o método de ensaio imunoenzimático.	3B
Musabak et al.^16^	Investigar a relação dos níveis séricos de IL-18 com a atividade da doença e as apresentações clínicas da DB.	Sessenta pacientes com DB e 20 controles saudáveis foram incluídos no estudo. Os pacientes foram agrupados como portadores de doença ativa ou inativa.	3B

DB = doença de Behçet; IL = interleucina; ISG = *International Study Group*; ISGBD = International Study Group for Behçet Disease; LCR = líquido cefalorraquidiano. 2C: Observação de resultados terapêuticos; 3B: Estudo caso-controle (Níveis de evidência científica segundo a classificação de *Oxford Centre for Evidence-Based Medicine*).

## DISCUSSÃO

A DB é uma vasculite sistêmica caracterizada por aftas orais dolorosas e recorrentes, úlceras genitais, lesões oculares e cutâneas (Tabela 2). O envolvimento vascular é observado em até um terço dos casos, podendo acometer vasos de todos os tamanhos, tanto do sistema arterial quanto do venoso, levando a oclusões e/ou formação de aneurismas^12^. Os achados anatomopatológicos mais observados são infiltrados de linfócitos e neutrófilos, que resultam em lesão do endotélio vascular.

**Tabela 2:** Frequência de lesões clínicas na doença de Behçet.

	**Frequência (%)**
Lesões orais	95
Manifestação neurológica	10-50
Manifestação gastrointestinal	5-60
Manifestação vascular	5-30
Manifestação cardíaca	1-5
Manifestação pulmonar	1-8

Entre os artigos de base desta revisão, houve uma notável heterogeneidade em relação às causas da DB. A etiologia ainda é incerta, porém certamente multifatorial, mas pode ser classificada em três principais e mais relevantes causas: genética, inflamatória e infecciosa. Esses três fatores inter-relacionados estão demonstrados na Figura 2, através do mecanismo imunopatológico da DB.

**Figura 2:** Diagrama do mecanismo imunopatológico da doença de Behçet - o alelo HLA-B51 associado às infecções medeiam a patogenia da doença de Behçet. Tais gatilhos ativam um sistema imunológico inadequadamente capazes de produzir citocinas e, consequentemente, células reativas, lesionando, por fim, os vasos. IL = interleucina; IFN = interferon; TNF = fator de necrose tumoral.

### Fator genético

Desde os primeiros relatos da possível relação do alelo HLA-B51 com a DB, embora essa associação ainda se mantenha indefinida, houve uma série de estudos acerca do fato^2^. Muitos deles relataram prevalência mais alta do alelo HLA-B51 em pacientes portadores de DB, além da associação com expressões mais complexas e graves das manifestações clínicas e desfechos piores do envolvimento ocular ou neurológico^10^. Ademais, foi demonstrado que o alelo HLA-B51 predomina em homens e está associado a prevalências moderadamente mais altas de úlceras genitais, manifestações oculares e cutâneas, além de alterações na coagulação e no endotélio, aumentando o risco de desenvolvimento de aneurismas e eventos trombóticos^9^^,^^12^.

Um estudo retrospectivo de 2004 a 2016 do Departamento de Medicina Interna e Endocrinologia do Hospital Universitário Fattouma Bourguiba, na Tunísia, mostrou a presença do alelo em 15,92% dos pacientes portadores da DB^13^. Neste mesmo estudo, realizado em 213 pacientes, também foram demonstrados dados importantes da DB como a presença das lesões orais em 98,6% dos pacientes em acompanhamento e 30% de acometimento vascular, sendo, portanto, importantes no diagnóstico da doença.

No entanto, os fatores genéticos não podem explicar totalmente a patogênese desta doença. A hipótese do alelo HLA-B51 associada à diversos gatilhos ambientais, como infecções bacterianas e virais, por exemplo, levando a uma exacerbação do processo imune e da resposta inflamatória parece ser mais sensata^17^.

Entretanto, a ausência do gene HLA-B51 em cerca de 80% dos pacientes, sugere que outros fatores também devem ser avaliados na gênese da DB^10^^,^^18^.

### Processo inflamatório e imunológico

A resposta inflamatória e o processo imunológico são incertos e complexos e podem levar a alteração da cascata de coagulação e lesão endotelial, favorecendo a formação de trombos, êmbolos e aneurismas^19^. Recentemente, a imunopatogênese e o envolvimento das células imunes e adaptativas e citocinas na gênese da DB vêm sendo debatidas^10^.

As células *natural killer* (NK), as principais da imunidade inata, não apenas desempenham um papel citotóxico nas células infectadas, mas também regulam as funções de outras células do sistema imune secretoras de citocinas, dentre elas TNF‐α, interferon (IFN) γ e α e interleucinas (IL) 2, 4 e 10^7^.

Os neutrófilos também desempenham um papel fundamental na resposta imune inata e caracterizam-se por serem a primeira linha de defesa contra doenças infecciosas. Estudos demonstraram que um dos principais mecanismos na patogênese da DB é a ativação de neutrófilos com aumento da quimiotaxia e da geração de radicais superóxido. Além disso, um estudo evidenciou que os neutrófilos nos pacientes com a DB exibem alta ativação intrínseca que pode estar associada à presença do gene HLA-B51 e estão envolvidos na infiltração perivascular das lesões da DB vascular^14^.

As respostas adaptativas são representadas na doença pelas células T de padrão de resposta Th 1 e 17, que desempenham um papel importante na patogênese da doença^7^. A resposta imune mediada por células Th1 desempenha um papel fundamental na patogênese da DB, e sua prevalência é significativamente maior em pacientes com DB ativa do que naqueles em pacientes com DB inativa^7^. A Tabela 3 sintetiza como essas células atuam na patogênese da doença, além do papel das citocinas envolvidas.

**Tabela 3:** Principais células envolvidas na patogênese da doença de Behçet.

**Célula NK**	Nas doenças autoimunes, a frequência das células NK diminui e sua citotoxicidade é prejudicada. Alguns estudos demonstraram que o número de células NK em pacientes com DB diminuíram, comprometendo a imunidade.
**Neutrófilos**	Neutrófilos podem danificar células e tecidos hospedeiros, sendo, assim, o fator desencadeante da inflamação, que, por sua vez, desencadeia a resposta imune.
**Células Th1**	As células Th1 que produzem IFN-γ que ativam macrófagos, responsáveis pela imunidade mediada por células a patógenos intracelulares e estão associados a muitas doenças autoimunes específicas de órgãos, incluindo a DB.
**Células Th17**	A frequência de células Th17 circulantes em pacientes com DB ativa é significativamente maior do que nos mesmos pacientes no estágio de remissão.

DB = doença de Behçet; IFN = interferon; NK = *natural killer*.

### Infecções

Infecções bacterianas ou virais podem servir de gatilhos para o desenvolvimento da doença em indivíduos portadores do alelo HLA-B51^12^. Os microrganismos mais associados à DB são o herpes vírus simplex ou *Streptococcus* ssp, porém também há descrições de outros, como parvovírus B19, *Helicobacter pylori*, citomegalovírus, Epstein-Barr vírus, herpes zoster vírus e *Staphylococcus aureus* 2^,^^7^.

Essas infecções estão associadas à inflamação sistêmica que, em indivíduos geneticamente suscetíveis, pode desencadear uma resposta imune inadequada e exacerbada e provocar lesão endotelial. Esse processo é demonstrado na Figura 2.

### O papel das citocinas inflamatórias

A imunopatogênese da DB foi amplamente estudada e várias células do sistema imunológico e citocinas podem estar envolvidas, sobretudo as pró-inflamatórias, que aceleram o prejuízo da função endotelial^7^. Um estudo evidenciou que muitas dessas citocinas inflamatórias foram encontradas, de forma aumentada, em soro de pacientes com DB, e, por causa dessas evidências, os antagonistas de TNF-α e IFN-α demonstraram boa eficácia e são os agentes usados no tratamento da DB^7^.

Na Figura 2, primeiramente, observa-se uma associação da doença com o alelo HLA-B51, que demonstra risco maior para a DB. Os agentes infecciosos também podem servir de gatilho para uma resposta imunológica anormal. Ambos podem desencadear a hiper-reatividade das células T e neutrófilos^14^. Os linfócitos T respondem a infecções virais e bacterianas, tendendo, assim, para um padrão de resposta Th1 e Th17, que produzem mediadores pró-inflamatórios, como IL-2, IL-6, IL-8, IL-12, IL- 18, IL-21, TNF-α e IFN-γ^7^. Vale ressaltar como algumas delas se relacionam com a DB:

IL-6: a produção excessiva de IL-6 está relacionada a doenças inflamatórias autoimunes e crônicas. Foi relatado aumento de IL-6 no líquido cefalorraquidiano em pacientes com envolvimento neurológico na DB^15^.

IL-18: desempenha um papel importante na resposta imune das células Th1. As funções da IL-18 são promover a produção de IFN-γ ativando células NK e induzir a atividade citotóxica. Os níveis de IL-18 foram mais altos em todos os subgrupos de pacientes quando comparados aos controles saudáveis, demonstrando a correlação dessa citocina com a atividade da doença^16^.

IFN-γ: as células Th1 que produzem IFN-γ ativam macrófagos e estão associadas a doenças autoimunes, incluindo a DB^7^. Em um estudo que avaliou o papel das citocinas na uveíte da DB, foi relatado que o nível de IFN-γ no humor aquoso dos pacientes com DB foi significativamente maior do que nos pacientes com DB, porém sem uveíte^11^.

TNF-α: desempenha um papel fundamental na indução e manutenção da inflamação na resposta autoimune, além de estar envolvido em vários processos fisiológicos e patológicos, como o início da inflamação, imunorregulação e proliferação celular^8^. Nas doenças inflamatórias, o TNF-α é produzido principalmente por macrófagos, células T, células B, neutrófilos, células NK e células endoteliais. Os níveis séricos de TNF-α estão aumentados em pacientes com DB ativa^7^.

IL-21: é uma citocina da família das IL-2, que promove expansão de células T CD8^+^ efetoras e pode ativar células NK^7^. Há evidências do papel crítico da IL-21 na condução de lesões inflamatórias na DB, promovendo Th17 efetores e suprimindo as células T reguladoras, representando um alvo promissor para uma nova terapia na DB^20^.

### Acometimento vascular na DB

A vasculite na DB é predominantemente neutrofílica, acometendo todas as camadas do vaso e *vasa vasorum*, podendo apresentar espessamento fibroso e infiltrado inflamatório inespecífico em sua fase tardia^5^. Associado à vasculite, há também presença de hipercoagulabilidade, com formação excessiva de trombina, diminuição de fibrinólise, hiperatividade plaquetária e formação dos complexos plaqueta/neutrófilos^2^. Todas essas alterações podem favorecer a formação de trombose. O processo inflamatório na parede do vaso pode causar destruição das fibras elásticas, levando à necrose transmural de parede das grandes artérias musculares, com consequente formação de aneurismas^5^.

### Tratamento

Apesar das opções de terapias tradicionais com uso de glicocorticoides e imunossupressores, há poucas evidências quanto à eficiência desses tratamentos na DB^1^. Os antagonistas do TNF-α e IFN têm demonstrado boa eficácia e são os agentes de primeira linha usados para melhorar o prognóstico da DB. O tratamento dos pacientes com antagonistas do TNF-α (Infliximabe, Etanercepte, Adalimumabe) se baseia no controle da resposta inflamatória, eficiente em manifestações graves e refratárias da DB^7^. A terapia com IFN, sobretudo o INF-α, tem demonstrado benefícios no tratamento habitual da doença, com atividades antiviral, antitumoral e imunomoduladora eficazes no manejo da DB^1^. Porém, ainda há escassez de dados sobre essas abordagens terapêuticas ideais e falta de marcadores laboratoriais informativos para monitorar a progressão da doença^21^.

## CONCLUSÃO

A patogênese da DB ainda não é bem esclarecida. Entretanto, estudos têm demonstrado que alguns gatilhos, como infecções bacterianas e virais, e predisposição genética (presença do alelo HLA-B51) podem iniciar e interferir no desenvolvimento da DB, pela ativação exacerbada do sistema imunológico, resultando da produção de inúmeras citocinas capazes de gerar células reativas responsáveis pelas lesões nos vasos e hipercoagulabilidade. Contudo, estudos mais detalhados são necessários para elucidar melhor os mecanismos envolvidos no processo imunopatológico da DB.
